# Mapping the categories of the Swedish primary health care version of ICD-10 to SNOMED CT concepts: Rule development and intercoder reliability in a mapping trial

**DOI:** 10.1186/1472-6947-7-9

**Published:** 2007-05-02

**Authors:** Anna Vikström, Ylva Skånér, Lars-Erik Strender, Gunnar H Nilsson

**Affiliations:** 1 Department of Neurobiology, Care Sciences and Society, the Center for Family and Community Medicine, Karolinska institutet, SE-141 83, Huddinge, Sweden

## Abstract

**Background:**

Terminologies and classifications are used for different purposes and have different structures and content. Linking or mapping terminologies and classifications has been pointed out as a possible way to achieve various aims as well as to attain additional advantages in describing and documenting health care data.

The objectives of this study were:

• to explore and develop rules to be used in a mapping process

• to evaluate intercoder reliability and the assessed degree of concordance when the 'Swedish primary health care version of the International Classification of Diseases version 10' (ICD-10) is matched to the Systematized Nomenclature of Medicine, Clinical Terms (SNOMED CT)

• to describe characteristics in the coding systems that are related to obstacles to high quality mapping.

**Methods:**

Mapping (interpretation, matching, assessment and rule development) was done by two coders. The Swedish primary health care version of ICD-10 with 972 codes was randomly divided into an allotment of three sets of categories, used in three mapping sequences, A, B and C. Mapping was done independently by the coders and new rules were developed between the sequences. Intercoder reliability was measured by comparing the results after each set. The extent of matching was assessed as either 'partly' or 'completely concordant'

**Results:**

General principles for mapping were outlined before the first sequence, A. New mapping rules had significant impact on the results between sequences A - B (p < 0.01) and A - C (p < 0.001). The intercoder reliability in our study reached 83%. Obstacles to high quality mapping were mainly a lack of agreement by the coders due to structural and content factors in SNOMED CT and in the current ICD-10 version. The predominant reasons for this were difficulties in interpreting the meaning of the categories in the current ICD-10 version, and the presence of many related concepts in SNOMED CT.

**Conclusion:**

Mapping from ICD-10-categories to SNOMED CT needs clear and extensive rules. It is possible to reach high intercoder reliability in mapping from ICD-10-categories to SNOMED CT. However, several obstacles to high quality mapping remain due to structure and content characteristics in both coding systems.

## Background

Terminologies, concept systems and classifications are used for different purposes in health care and have different structures and content. Classifications are used mainly for statistical and reimbursement purposes. Terminologies are often used to describe clinical 'input' data within Electronic Health Record (EHR) -systems and are based on national or international terminology standards [1], or are developed locally or by vendors [2]. Concept systems or reference terminologies such as SNOMED CT are described as an international effort to produce and enhance a standard global clinical terminology, with the aim of providing a common language that enables a consistent way of indexing, storing, retrieving, and aggregating clinical data across specialities and sites of care [3]. With respect to its original purpose, a coding system such as the International Classification of Diseases (ICD-10) has shortcomings when scaling up for re-use for multiple purposes, such as in health care record systems, whereas reference terminologies directly address these scaling and re-use issues [4].

Linking or mapping terminologies and classifications has been pointed out as a possible way to accomplish different goals of classifications and terminologies as well as to attain additional advantages in describing and documenting health care data. [5,6]. The concept 'mapping' is described as 'linking terminology content between two schemes' [7]. Mapping can be done with entirely different methods comprising more or less automated procedures, lexical matching, concept matching and structural matching [8-13]. The importance of rules that are outlined in the mapping process has been described [8,12]. Maps developed between terminologies and classifications are designed differently based upon the intended use of the mapped data. Mapping for reimbursement purposes, where several rule-based instructions for coding would need to be incorporated, is different from mapping for epidemiological purposes [6].

The ICD-10, was endorsed by the 43d World Health Assembly in May 1990 and came into use in WHO Member States starting in 1994. The ICD has become the international standard diagnostic classification for all general epidemiological purposes and many health management purposes. It is used to classify diseases and other health problems recorded in many types of health and vital records including death certificates and hospital records. [14]. ICD has developed as a practical classification that includes a number of compromises based on aetiology, anatomical site, circumstances of onset, etc. [15]. ICD-10 has rules for coding; for example, the chapters have different priorities: chapters XV 'Pregnancy, childbirth and the puerperium' and XVI 'Certain conditions originating in the perinatal period', have the highest priority; and chapters I 'Certain infectious and parasitic diseases', II 'Neoplasms', and XVII 'Congenital malformations, deformations and chromosomal abnormalities' have higher priority than the chapters related to organ systems [16].

The terms 'include' and 'exclude' are also used to clarify what should and what should not be a part of a category. 'Exclude' is followed by a reference to another code. [16]. The categories including the word 'Other' in ICD-10 are residual categories for conditions that cannot be allocated to the more specific categories [15]. ICD-10 is not considered entirely suitable for primary care [17]. In Sweden, a primary health care version of ICD-10 has been developed that has the acronym KSH97-P [18].

The Systematized Nomenclature of Medicine, Clinical Terms (SNOMED CT) is the world's largest concept-based concept system with more than 300,000 concepts, 700,000 descriptions (terms) and 900,000 relations [19]. It was developed from earlier SNOMED versions and the Read Codes from the UK, and has one common concept model [20]. It has been suggested that concept systems such as SNOMED CT have a large volume and a granularity that are unsuited to the administrative purposes for which a classification is designed [5]. SNOMED CT concepts are mapped to categories in some classifications, for example ICD-9-CM in the US edition and ICD-10 in a UK-edition. SNOMED CT and ICD-10 are also mapped into the Unified Medical Library System (UMLS) [10]. Intercoder reliability was measured when locally used terms were coded to SNOMED CT concepts, with a Kappa value of 0.53 and a 58% matching rate when not correcting for errors [12]. When coding ophthalmology concepts to SNOMED CT, the intercoder reliability was 35 % (three coders) and 43 % (two coders) [21]. There is no 'gold standard' for matching rules between ICD-10 and SNOMED CT. It has been suggested that cross-mappings between SNOMED CT and classifications like ICD-10 should maximise the value of the clinical data and the benefits of an EHR-system [5]. The majority of concepts in SNOMED CT are 'primitive' - 85 % of the active concepts. [Personal communication, CAP 200611120]. A concept is primitive when its modelling (attributes and parents) does not fully express its meaning. 'Primitive' concepts do not have the unique relationships needed to distinguish them from their parent or sibling concepts, whereas 'fully defined' concepts can be differentiated from their parent and sibling concepts by virtue of their relationships. Some concepts should remain primitive [22].

Therefore it would be of interest to examine what level of intercoder reliability can be reached using a manual mapping process when mapping a subset of ICD-10 (KSH97-P) to SNOMED CT, and to determine which mapping rules are necessary in the process. Another aim would be to obtain better knowledge of the characteristics of the systems that need to be addressed when creating and using a mapping from ICD-10/KSH97-P to SNOMED CT.

The objectives of this study were

• to explore and develop rules to be used in a mapping process

• to evaluate intercoder reliability and the assessed degree of concordance when the Swedish primary health care version of ICD-10 is matched to SNOMED CT.

• to describe characteristics in the coding systems that are related to obstacles to high quality mapping.

## Methods

### Coders

Mapping was done by two coders (YS, AV). One of the coders is a primary health care physician (YS) and the other is a health informatician (AV), and both have broad experience in the area of terminology.

### SNOMED CT

The Clue system version, 5.5.0133, was used for browsing in SNOMED CT, and the versions of SNOMED CT that were used were from January and July 2006 [23]. The Clue system shows the concept, concept id, concept status (for example, 'current' or 'limited'), concept relations, and descriptions.

### KSH97-P

KSH97-P is a subset of ICD-10 categories. KSH97-P consists of a total of 972 categories in the 2004 version, out of which 611 categories correspond to one single ICD-10 category, and 361 categories are constructed as a cluster of ICD-10 categories with a new code name (here: P-categories). One example of a P-category is 'Nutritional deficiency, unspecified', which has 20 clustered categories from ICD-10 on a triadic alphanumeric code level. Each category in KSH97-P has a connection to one of 21 chapters in ICD-10. One of the chapters, chapter XXI, 'Factors influencing health status and contact with health services', contains categories initially labelled 'Z', (here: Z-categories). In KSH97-P categories, as in the Swedish version of ICD-10, 'and' should be interpreted as 'and/or', according to classification rules [16]. Several sources were used for the KSH97-P. One source was a file with the master KSH97-P category code and text in Swedish, and suggested English translations [24]. These categories matched the Swedish translation of ICD-10. Another source was an IT-system called 'Visaren', which showed the corresponding ICD-10 categories both on a chapter and a category level, as well as the 'exclude' rules and a 'recommended term' for each category that was a 'short term'[25].

### Translation

The English version of ICD-10 and the Swedish and English versions of Medical Subject Headings (MeSH) were used for general translation purposes [26].

### Mapping and assessment

The coders participated in two half-day seminars aimed at familiarising them with the different IT-systems used for browsing and the structures of the different coding systems. The 972 categories in KSH97-P were randomly divided into an allotment of three sets of categories with different content: A (n = 323), B (n = 326) and C (n = 323). Mapping was done independently by the coders in three sequences using the three different sets of categories. The mapping process comprised four activities:

1. **Interpreting**: which means that each coder analysed the meaning of concepts and categories including, when applicable, translations of keywords and search terms from Swedish to English.

2. **Matching: **which means that each coder matched one KSH97-P category to one SNOMED CT concept. Categories with no match in SNOMED CT were marked as '0' (none found).

3. **Assessing: **which means that each coder assessed every matched concept-category pair on how well they matched. The assessment scale used was 'partly concordant' (1) for concepts that approximated the category meaning, and 'completely concordant' (2) if the concept completely captured the meaning of the category. The main reason for the assessment was to prepare for further analysis of mapping results and analysis of the concept systems. A supplementary assessment of the 'partly concordant' concept-category pairs was done independently by the coders after the mapping of all three sequences, as they were assigned into three different groups: 'target (SNOMED CT) more specific than source (KSH97-P)' (a), 'target less specific than source' (b), and 'imprecise but neither more nor less specific'(c), using a categorisation from the SNOMED cross-mapping method [19].

4. **Rules **development, which means that each coder saw the need for and suggested new rules and decided upon rules in consensus with the other coder.

Few references were found regarding rules used for mapping from classifications to SNOMED CT. Many of the rules could have been formulated before the mapping process started, but due to lack of knowledge of the concept systems, most of the rules had to be created during the process. The rules were focused on reaching better intercoder reliability and qualitatively better mapping. Each coder added comments on the concept-category pair she chose for use when developing mapping rules.

### Intercoder reliability and analysis of obstacles

When the coders chose the same SNOMED CT concepts for one KSH97-P category, these were called 'equally chosen concepts'. This was measured as intercoder reliability by comparing the results after each of the three sequences, A, B and C. The reasons for different choices of concepts were analysed and initially divided into two major groups: a. misunderstandings or human errors, and b. structural and content factors in the different coding systems. The structural and content factors as obstacles to high quality mapping were analysed both statistically and qualitatively. A comparison was made between the intercoder reliability concerning categories in KSH97-P that corresponded to one single ICD-10 category and the P-categories constructed as a cluster of ICD-10 categories. The assessment of concordance between every matched concept-category pair was also measured.

### Statistics

The number and percentage share of matching results were calculated. Logistic regression was used, with the outcome variableindicating 'equally chosen concepts', to analyse if there were any significantdifferences betweensequences A, B and C. Pearson's Chi-square test was used when analysing results between the clustered ICD-10 categories (P-categories) and the non-P categories. Cohen's Kappa (K) and the percent agreement were used to measure the intercoder reliability of assessments. Suggested interpretations of K values are < 0.20 = poor, 0.21–0.40 = fair, 0.41–0.60 = moderate, 0.61–0.80 = good, and 0.81–1.00 = very good [27]. The percentage share was calculated based on the rate of equally chosen categories in each chapter of ICD-10.

## Results

### Rules

A general principle or rule for mapping that was outlined before the first sequence, A, was to have a concept oriented perspective. This refers to attaining knowledge of the definitions and explanations of the meaning of concepts and categories in each coding system on a higher level than category text and description; for example, examining parent and child concepts related to a concept in SNOMED CT and also examining all ICD-categories connected to a KSH97-P category. Another rule was not to use 'limited' concepts in SNOMED CT, defined as 'concepts of limited clinical value, as they are based on a classification concept or an administrative definition. Concepts with this status are still valid for current use and are considered active' [19]. This was an important rule, as there were many exact lexical matches to ICD-10 categories that were limited concepts. New rules were developed after sequences A and B (table 1).

### Intercoder reliability

The intercoder reliability (the percentage share of SNOMED CT concepts to KSH97-P-categories that were equally chosen by the coders) increased from 69 % to 83 % when adding mapping rules after sequences A and B (table 2). Logistic regression showed a significant difference between sequences A - B (p = 0.01), and sequences A - C (p = 0.001), but not between B and C (p = 0.055). The intercoder reliability for the entire set (A, B and C) of KSH97-P categories and SNOMED CT concepts was 77 %.

The P-categories, which were clustered ICD-categories, varied between sequences A, B and C (table 2). The P-categories were randomly distributed in the allotment of three sets of categories as follows: sequence A, 130, sequence B, 122, and sequence C, 109. The percentage share of equally chosen P-categories differed significantly between A and B when Pearson's Chi square was used (p = 0.047), as well as between A and C (p = 0.004), but not between sequences B and C (p = 0.334). The rate of equally chosen P-categories for the entire set was 68% as compared to the rate for non-P-categories, which was 82 %.

**Figure 1:**
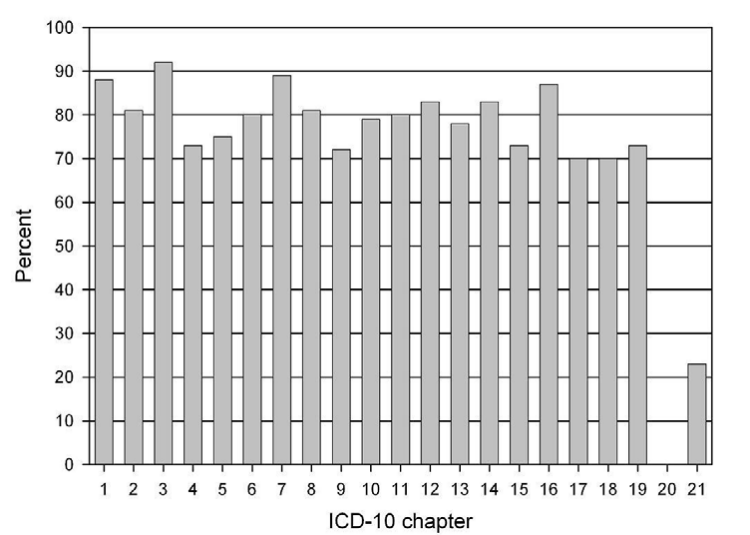
**Equally and not equally chosen concepts matched to categories in different chapters of ICD-10**. Chapter 21 with the Z-codes had a 23% rate of equally chosen concepts. An equally chosen category = when the two coders matched the same SNOMED CT concept to one KSH97-P category.

The Z-categories from chapter XXI in ICD-10 had a 23% rate of equally chosen categories, which was lower compared to other chapters (figure 1).

### Assessment and errors

The subjective assessment by each coder of every matched concept-category pair and its concordance was 85.3% or 829 (Coder 1), and 87.9% or 854 (Coder 2), respectively, for (2) 'completely concordant', and for (1) 'partly concordant' it was 13% or 127 (coder 1), and 11.7% or 114 (coder 2), respectively. The intercoder reliability for the coders' assessments reached 89%, which was moderate (K = 0.49). The assignment of the 'partly concordant' concept-category pairs into three groups is shown in table 3.

Non equally chosen SNOMED CT concepts due to human factor errors and structural and content dependent factors in the coding systems are shown in table 4. Examples of human errors were missing characteristics in a concept, such as 'acute' in 'acute otitits media', and not following the mapping rules.

### Structural and content dependent factors in the coding systems

SNOMED CT contains more specialized concepts than ICD-10. This led to choices of different but related or similar concepts from SNOMED CT, considered as concepts in good agreement with a KSH97-P category. Examples of such cases are shown in table 5.

Other issues related to SNOMED CT were difficulties in interpreting meaning in concepts that lacked term or description transparency, textual definitions or were not fully defined ('primitive').

The differences between the SNOMED CT concepts 'system', 'organ' and 'tract' have no corresponding groupings in ICD-10. One example of this is 'congenital anomaly of digestive system', 'congenital anomaly of digestive tract' and 'congenital anomaly of digestive organ', which in ICD-10 is 'congenital malformation of digestive *system*' and in the Swedish version is translated as '*organ*'.

The rules in SNOMED CT for using the terms 'abnormality', 'anomaly', 'deformity' and 'malformation' together with 'congenital' were not clear.

In ICD-10/KSH97-P, the predominant reasons for non equally chosen concepts were difficulties in interpreting the meaning of the categories. One example originates from the Z-categories in chapter XXI: Z712 'Person consulting for explanation of investigation findings'. This category does not distinguish between persons and patients, or between patients and contacts with health care, which are separate concepts. Another example is 'Blindness and low vision' where 'and' should be interpreted as 'or', according to classification rules [16]. 'And/or' is a common expression in SNOMED CT that is not present in KSH97-P or ICD-10.

Another type of category in ICD10/KSH97-P that was difficult to match comprised categories that begin with the qualifier value 'other', and 'other specified'; for example, 'Other complications of surgical and medical care, not elsewhere classified', and 'Other specified general symptoms and signs', as there are no concepts for diseases or findings, except the 'limited' concepts, that begin with 'other' in SNOMED CT. This is similar to the 'exclude rules', which are not present in SNOMED CT.

There were also difficulties in ICD10/KSH97-P with 'aggregated' categories where more than two organs, systems or other objects were present that did not have a match in SNOMED CT; for example, 'Neoplasm of uncertain or unknown behaviour of middle ear and respiratory and intrathoracic organs', and 'Abscess, furuncle and carbuncle of nose'.

## Discussion

New mapping rules had a significant impact on the results between sequences A - B and A - C. Mapping from ICD-10-categories to SNOMED CT needs clear and extensive rules. The intercoder reliability in our study reached 83%. The obstacles to high quality mapping were mainly differences in agreement between coders due to both structural and content factors in SNOMED CT and ICD-10/KSH97-P.

It can be questioned whether better mapping rules would have further improved the reliability, as there was no significant improvement between sequences B and C. Some of the rules are obvious, such as not using navigational concepts, and could have been outlined before the mapping process. The absence of documented references regarding mapping rules was one reason for designing a study where the rules were developed in a manual mapping process. The reason for not using 'limited' concepts that were based on a classification concept or an administrative definition was that they did not seem to be modelled into SNOMED CT like other concepts, as they were 'hanging in the end' of the hierarchies in SNOMED CT. They seemed to be less well defined and had terms in the descriptions that were not used for other concepts. Several of these concepts even had a similar concept in the hierarchy without limited status.

A study that mapped narrative parish nurse documentation (170 health records, 1607 interactions) into the Nursing Interventions Classification (NIC), which is included in SNOMED CT, initially yielded a moderate intercoder reliability as measured by K (0.53) and by percent agreement (58%). After correcting mapping errors, there was 68% agreement, and after discussion between coders the figure was 93% (K = 0.92) [12]. These results are lower than in our study, where an 83% reliability was attained without correcting errors. The reason for the lower figure may be that the study examined reliability in mapping from terms in nursing documentation to SNOMED CT/NIC, and not between two coding systems.

A study on intercoder reliability between three coders coding ophthalmology concepts to SNOMED CT and several other classifications showed a low level of agreement for exact matching between three coders (35%), and between two coders (43%). This was coding from ophthalmology case presentations selected from a publicly available journal, parsed into discrete concepts, and not mapping between coding systems [21].

In a study where common patient problems were automatically mapped to SNOMED CT and manually reviewed by two reviewers, the judgement regarding SNOMED CT was 91.8% with K = 0.49 [28], as compared to our study where the figure reached 83%.

There were several obstacles to achieving high quality mapping. Similarity or relatedness between concepts in SNOMED CT was found in our study to be one reason for different chosen concepts. An evaluation study found many 'similar concepts' in SNOMED CT by locating concepts that contained the same non-hierarchical relationships, as well as by searching with keywords, which is similar to the method used in our study [29]. Relatedness refers to human judgements regarding the relatedness of pairs of concepts [30].

Another factor of importance concerns the clinical usefulness of such related concepts. The ophthalmology coding study reported that coders found 'semantically equivalent' concepts in SNOMED CT, judged to have no clinically significant difference in meaning, and that this may decrease intercoder reliability in clinical practice [21]. Concepts that are so closely related that no obvious clinical distinction can be found cannot be expected to be used in a reliable way in clinical practice. Also, the absence of rules for selecting a 'finding' or a 'disorder' concept as illustrated in Table 5 is a factor of importance. Our study found that the presence of many related concepts in SNOMED CT was one of the reasons the coders chose different concepts. This raises the question of the clinical usability, with respect to intercoder reliability, of such an extensive concept system as SNOMED CT.

There were also several obstacles in ICD-10/KSH97-P to attaining high quality mapping. For example, the classification structure in ICD-10 with the 'exclude' rule is not present in SNOMED CT. Every disease or morbid condition must have a well defined place [15]. 'Gout', for example, can be classified under arthritis or metabolic disorders, but not under both [4]. The absence of these rules makes it unsuitable to replace a classification like ICD-10 with a concept system like SNOMED CT, as they have different purposes.

In ICD-10, the axes of the classification are not consistent [4], while the concepts in SNOMED CT are modelled consistently into one concept model. There are many concept relations in SNOMED CT that represent relations existing 'in clinical thinking' and that are not dependent on the principles of grouping chapters in ICD-10 - a concept can have 'parent concepts' in more than one domain. One example of this is 'Noise effects on inner ear' that have an 'is_a' relation to 'Ear finding', which in turn have 'is_a' relations to both 'Ear, nose and throat finding' and the concept 'Effect of exposure to physical force'. These multiple axes or hierarchies make it possible to access a concept through all reasonable hierarchic paths [31], and can therefore be used to relate 'large' or more general concepts to categories that are present in many different chapters in ICD-10. It can, for example, be easier to find and gather information from categories related to heart disease that are currently found in at least 13 different chapters in ICD-10 [4].

In categories in KSH97-P with 'and', this should be interpreted as 'and/or'. That rule was not followed in this study, as the aim was to find matches in SNOMED CT to every object present in a category. If 'and' means 'and/or' in all categories in ICD-10/KSH97-P, it is not obvious why the objects should be aggregated in a category the way they are. An example of this is 'Somnolence, stupor and coma'. This absent 'and/or' rule generates ambiguity both regarding interpretation of the meaning of a category and the correct way to map to SNOMED CT concepts.

Another example of difficulties in interpreting category meaning in KSH97-P is the so-called 'recommended term' for each category, which is a 'short term' that often narrowed and sometimes confused the concept meaning. One example of that is the short term 'Poisoning by drugs' that refers to 'Poisoning by drugs, medicaments and biological substances', which is a wider category.

The method of combining several SNOMED CT concepts (post coordination) was not used. It is most likely possible to obtain a higher rate of equally chosen categories between SNOMED CT and KSH97/ICD-10 if post coordination is used, as shown in a study where the use of compositional concepts provided significant improvement in the content coverage of common problem statements by SNOMED CT (92.3% vs. 51.4% [28]. However, post coordination demands advanced knowledge of the post coordination rules, which is the main reason the method was not used in this study. The assignment of the 'partly concordant' concepts into three groups showed that both coders found a high percentage of the chosen SNOMED CT concepts to be more specific than the source (KSH97-P). This implies that a mapping from ICD 10 categories to SNOMED CT concepts would benefit from post coordination.

A limitation of this study is the use of an entirely manual mapping process using the Clue Browser. Manual browsing can lead to low recall. One of the reasons for using a completely manual mapping method in the present study was that KSH97-P is a small classification with 972 categories, which made it easy to perform the manual method and also provided valuable insight into the coding systems. Also, SNOMED CT is not translated into Swedish, which is required when automated lexical mappings are used. The necessary translations have complicated the matching process. The English terminology used in ICD-10 and the Swedish classifications based on ICD-10 are somewhat different from the translation and terminology of MeSH and Swedish MeSH. In this study, several concepts in SNOMED CT had terminology that was more similar to that of MeSH than to ICD-10, but the opposite was also found.

The translation issues in this study show that a possible translation of SNOMED CT into Swedish should also include deliberate decisions regarding rules that conform or do not conform to earlier translations of ICD-10 and other classifications translated to Swedish.

It has been suggested that cross-mappings between SNOMED CT and classifications like ICD-10 should maximise the value of the clinical data and the benefits of an EHR system. Interpretation of epidemiological statistics could benefit from the use of SNOMED CT when analysing diagnostic categories from ICD-10 in patient records in primary health care.

## Conclusion

Mapping from ICD-10-categories to SNOMED CT needs clear and extensive rules. It is possible to reach high intercoder reliability in mapping from ICD-10-categories to SNOMED CT. However, several obstacles to high quality mapping remain due to structure and content characteristics in both coding systems. A mapping from ICD 10 categories to SNOMED CT concepts would benefit from post coordination.

## Competing interests

The author(s) declare that they have no competing interests.

## Authors' contributions

AV wrote the manuscript and participated in the acquisition of data and in the design of the study. YS participated in the acquisition of data, the design of the study, and in drafting and revising the manuscript. L-E S contributed to the design of the study, and to revising the manuscript. GN contributed to the design of the study and to drafting and revising the manuscript.

## Pre-publication history

The pre-publication history for this paper can be accessed here:



## Figures and Tables

**Table 1:** Mapping rules after sequences A, B and C

**Rule**	**Examples and explanations**
To give priority to concepts from the 'disorder' or 'finding' hierarchy in SNOMED CT over 'morphological structure', 'body structure' or 'context depending category'/'situation concepts'	Choosing 'malignant lymphoma (disorder)' instead of 'malignant lymphoma (morphologic abnormality)
Not to use 'navigational' concepts	'Navigational concepts' is a child to the top-level concept 'Special concept'. These concepts are not part of active clinical terminology.
To thoroughly analyse if the Z-categories in chapter XXI of ICD-10 refer to procedures or states	Chapter XXI refers to 'factors' and 'circumstances other than a disease, injury or external cause' but does not state that many of the categories in the chapter seem to be referring to procedures; for example, 'Z 32-P Pregnancy examination'. Our decision was to map categories referring to procedures to procedure-concepts in SNOMED CT.
To consider the 'exclude' rule in ICD-10 as a rule that does not exist in SNOMED	The classification rule that 'excludes' categories both on a category and a chapter level is not present in SNOMED CT
To adhere to strict rules of priority regarding master data information sources for the classification categories in KSH97-P	There were several sources regarding master data in both English and Swedish that had to be ranked, since the sources were sometimes in conflict.
To give priority to order of terms in the original master category text in cases where the concepts were partly matched to SNOMED CT	'Other disorders of kidney and ureter' where 'disorder of kidney' should be chosen if the whole category could not be covered in a SNOMED CT concept.

**Table 2:** Intercoder reliability between sequences A, B and C, and the number and percentage share of equally chosen SNOMED CT concepts matched to P-categories (clustered ICD-10 categories within KSH97-P).

	Sequence A (n = 323)	Sequence B (n = 326)	Sequence C (n = 323)
Equally chosen SNOMED CT concepts matched to all KSH97-P categories	223 (69%)	254 (78%)	269 (83%)
Equally chosen SNOMED CT concepts matched to P-categories (clustered ICD-10 categories within KSH97-P)	76 (59%)	86 (70%)	83 (76%)

**Table 3:** Extent of matching in the concept-category pairs assessed by each coder (YS and AV) and non found concepts in SNOMED CT.

**Concordance**	**Coder 1(YS)**		**Coder 2 (AV)**	
0 (non found)	16 (1.7%)		4 (0.4%)	
1 (partly concordant)	127 (13%)		114 (11.7%)	
1a. target (SNOMED CT) more specific than source (KSH97-P)		77 (60%)		75 (65%)
1b. target less specific than source		25 (20%)		20 (18%)
1c. imprecise but neither more nor less specific'		25 (20%)		19 (17%)
2 (completely concordant)	829 (85.3%)		854 (87.9%)	
Total	972 (100%)	127 (100%)	972 (100%)	114 (100%)

**Table 4:** Number of non equally chosen SNOMED CT concepts and human dependent errors.

	**Sequence A**	**Sequence B**	**Sequence C**	**Sequence A-C**
Non equally chosen SNOMED CT concepts due to human dependent errors	14	11	3	28
Non equally chosen concepts due to structural and content dependent factors in SNOMED CT and ICD-10/KSH97-P	86	61	51	198

**Table 5:** Examples of different but similar concepts in SNOMED CT chosen by the coders (YS and AV)

**KSH97-P category**	**SNOMED CT concepts chosen by coder 1 (YS)**	**SNOMED CT concepts chosen by Coder 2 (AV)**
Hallux valgus	Acquired hallux valgus (disorder)	Hallux valgus (disorder)
Disorder of back, unspecificed	Dorsopathies (disorder)	Disorders of back (disorder)
Malunion of fracture	Malunion and nonunion of fracture (disorder)	Disorder of fracture healing (disorder)
Plantar fascial fibromatosis	Plantar fascial fibromatosis (disorder)	Plantar fasciitis (disorder)
Menopausal and female climacteric states	Menopause finding (finding)	Female climacteric state, function (observable entity)
Effects of vibration	Effects of vibration (disorder)	Vibration syndrome (disorder)
Trigeminal neuralgia	Trigeminal nerve disorder (disorder)	trigeminal neuralgia (disorder)
Ischaemic chest pain	Ischemic chest pain (finding)	Angina (disorder)
Other difficulties with micturition	Difficulty passing urine (finding)	Dysfunctional voiding of urine (finding)
Tics	Tic (disorder)	Tic (finding)
